# Older Adults with Multi-Morbidity: Medication Management Processes and Design Implications for Personal Health Applications

**DOI:** 10.2196/jmir.1813

**Published:** 2011-06-29

**Authors:** Leah M Haverhals, Courtney A Lee, Katie A Siek, Carol A Darr, Sunny A Linnebur, J Mark Ruscin, Stephen E Ross

**Affiliations:** ^7^Division of General Internal MedicineSchool of MedicineUniversity of Colorado Anschutz Medical CampusAurora, COUnited States; ^6^School of PharmacySouthern Illinois University EdwardsvilleEdwardsville, ILUnited States; ^5^School of PharmacyUniversity of Colorado Anschutz Medical CampusAurora, COUnited States; ^4^Colorado Health Outcomes ProgramSchool of MedicineUniversity of Colorado Anschutz Medical CampusAurora, COUnited States; ^3^Wellness Innovation and Interaction LabDepartment of Computer ScienceUniversity of Colorado BoulderBoulder, COUnited States; ^2^Department of Health and Behavioral SciencesUniversity of Colorado DenverDenver, COUnited States; ^1^Colorado REAP (Research Enhancement Award Program) to Improve Care CoordinationVeterans Affairs Medical CenterDenver, COUnited States

**Keywords:** Older adults, medication management, health records, personal

## Abstract

**Background:**

Older adults often have multiple chronic problems requiring them to manage complex medication regimens overseen by various clinicians. Personal health applications (PHAs) show promise assisting in medication self-management, but adoption of new computer technologies by this population is challenging. Optimizing the utility of PHAs requires a thorough understanding of older adults’ needs, preferences, and practices.

**Objective:**

The objective of our study was to understand the medication self-management issues faced by older adults and caregivers that can be addressed by an electronic PHA.

**Methods:**

We conducted a qualitative analysis of a series of individual and group semistructured interviews with participants who were identified through purposive sampling.

**Results:**

We interviewed 32 adult patients and 2 adult family caregivers. We identified 5 core themes regarding medication self-management challenges: seeking reliable medication information, maintaining autonomy in medication treatment decisions, worrying about taking too many medications, reconciling information discrepancies between allopathic and alternative medical therapies, and tracking and coordinating health information between multiple providers.

**Conclusions:**

This study provides insights into the latent concerns and challenges faced by older adults and caregivers in managing medications. The results suggest that PHAs should have the following features to accommodate the management strategies and information preferences of this population: (1) provide links to authoritative and reliable information on side effects, drug interactions, and other medication-related concerns in a way that is clear, concise, and easy to navigate, (2) facilitate communication between patients and doctors and pharmacists through electronic messaging and health information exchange, and (3) provide patients the ability to selectively disclose medication information to different clinicians.

## Introduction

Medication self-management is essential to drug safety but remains a challenging issue to address. Many patients, particularly older adults, have problems understanding their medication regimens or remembering to take their medications despite the use of patient information sheets and pillboxes [1]. Older adults experience more comorbidity, which increases the complexity of medication regimens, the risk of nonadherence, and the likelihood of fragmented care [2,3]. As a result, preventable adverse drug events in ambulatory care are more common among older adults [4].

Paper-based personal health records (PHRs) improve medication self-management for older adults in care transitions [5]. In theory, a PHA could provide on-demand, personalized, authoritative information on health issues, enhance management of medication information, and improve communication with health professionals and caregivers wherever a person may be [6,7]. However, privacy [8-13], usability [14-16], and updating [16] issues are potential barriers to the adoption of PHAs by older adults and caregivers. To better understand these issues, as part of a user-centered design process, we employed qualitative research techniques to elucidate the medication self-management needs and strategies of older adults and their adult caregivers that could be addressed through effective PHA design.

## Methods

This study was conducted under the review and oversight of the Colorado Multiple Institutional Review Board.

### Inclusion Criteria

All participants had to meet the following inclusion criteria: English fluency, the ability to pass a brief cognitive screen (provide name, year of birth, age, and telephone number), and their agreement that they would consider use of a computer application to manage health information. Patients of interest were defined as persons 65 years old or older, taking at least 3 prescription medications as an outpatient, with 2 or more outpatient visits in the last year, and 1 or more chronic medical conditions. Participants could either be “patients of interest” or “adult family caregivers” (caregivers for spouses, family members, or friends) of patients of interest.

### Recruitment

We used a purposive sampling strategy to capture variations in education, ethnicity, income levels, and living arrangements. Participants were recruited from 4 sites in the metropolitan areas of Denver and Boulder, Colorado: (1) an academic hospital-based ambulatory geriatric clinic in Aurora, Colorado serving adults living in private homes, (2) a municipal senior citizen center in Denver, Colorado catering to the surrounding working-class community, (3) a small, independent-living residential facility in suburban metro Denver with middle-class clientele, and (4) an assisted/independent-living facility in Boulder, Colorado serving wealthier and more highly educated older adults. Recruitment was coordinated with facility directors, and research team members visited sites in person to explain the study and distribute flyers about it. Participants were recruited via these flyers or through word of mouth and were given a gift card for $25 to a local grocery store for participation in the study.

### Data collection

First, 2 exploratory focus groups were conducted (15 participants in total) to broadly survey medication management challenges and to refine the topic guide for subsequent individual interviews. Minor refinements to the topic guide were made after a pilot interview with 2 older adults. We conducted 1- to 2-hour interviews in the homes of 16 participants, and one interview took place in a hospital. Researcher observations were recorded and digital photographs were taken to document patient arrangements for medication management techniques. After qualitative analysis of the individual interviews, 2 confirmatory focus groups were conducted to test and refine the themes and conclusions that were derived. All interviews and focus groups used semistructured topic guides, and were audio-recorded using a digital recorder, transcribed for analysis by a transcriptionist, and reviewed for accuracy by the research assistant who conducted the interviews and focus groups. During all phases of data collection and analysis, an advisory board of health professionals reviewed protocols, topic guides, and interim findings. All interview and focus group guides are available in the multimedia appendices.

### Qualitative analysis

Data collection activities provided data in the form of field notes, photographs, and interview and focus group transcriptions. A member of the research team then systematically coded these using ATLAS.ti (6.0, Scientific Software Development GmbH, Berlin, Germany) using both a deductive approach based on the initial agreed-upon themes to look for and an inductive approach that allowed new themes to emerge from the data. Each primary code and its associated quotations were then reviewed and discussed by 3 members of the multidisciplinary research team. Discrepancies in analysis were discussed, revised, and synthesized into a core set of themes. Members of the research team identified quotes during analysis that were particularly illustrative of these themes.

## Results

Participants in focus groups and interviews were 32 older adult patients and 2 family caregivers. From this pool of participants, 15 participated in the exploratory focus groups, 17 participated in individual interviews, and 10 participated in the confirmatory focus groups. Five older adults participated in both exploratory focus groups and individual interviews; 3 older adults participated in both individual interviews and confirmatory focus groups. Demographic information was collected from individual interview participants only (Table 1).

**Table 1:** Demographics of interview participants^a^

		Older patients (n = 15)	Family caregivers (n = 2)
Age (mean years, range)		82, 73–90	53, 48–57
Race: white (n, %)		13, 87%	1, 50%
Ethnicity: Hispanic (n, %)		2, 13%	1, 50%
Gender: female (n, %)		9, 60%	2, 100%
Has computer access (n, %)		10, 67%	2, 100%
**Use of computer (n, %)**			
	Regular	5, 33%	2, 100%
	Rare	3, 20%	0, 0%
	None	7, 47%	0, 0%
Has Internet access (n, %)		8, 53%	2, 100%
**Use of Internet (n, %)**			
	Regular	4, 27%	2, 100%
	Rare	3, 20%	0, 0%
	None	8, 53%	0, 0%

^a^The only demographic data captured from focus groups was gender and is not included here.

There were 13 individual interviews, composed of 15 older patient participants and 2 family caregivers, which yielded more detailed information about medication management. All of the 15 older patient participants managed their own medications, and 5 also assisted a friend or spouse in managing their medications. In 4 of the 13 interviews, 2 participants were present, and in 3 of the 13 interviews, a participant was interviewed in the presence of the cognitively impaired person they cared for.

Our qualitative analysis identified 5 key concerns of older adults surrounding medication self-management: seeking reliable medication information, maintaining autonomy in medication treatment decisions, worrying about taking too many medications, reconciling information discrepancies between allopathic and alternative medical therapies, and tracking and coordinating health information with and between multiple providers.

### Seeking Reliable Medication Information

Participants most often sought medication information from pharmacists and clinicians, followed by the insert that comes with medications and the Internet. Other less frequent medication information sources cited were family, friends, reference books, and nurses. Information sources were selected based on accessibility and the type of information needed. The most readily available information (eg, the Internet and the medication insert) was not always seen as the most credible or useful. Participants tended to use various information-seeking strategies to get what they needed.

Pharmacists were generally the most trusted source of medication information, based on their knowledge of drugs and interactions. Participants who ordered prescriptions by mail felt that pharmacist consultation by toll-free number was trustworthy and convenient.

A majority of participants also named their doctor as a source for medication information. However, while doctors were highly valued for questions about general health issues, many participants were concerned that doctors chose medications without fully considering drug interactions or costs. Doctors were least likely to be consulted if a timely response was required. About half the participants said, unprompted, that they felt their doctor was too busy to address their medication information needs. Overall, while some participants described long-term, trusting relationships with doctors, many felt that answers to medication questions during rushed visits were unclear. For follow-up questions, many were frustrated about not being able to talk to their doctor on the phone. Although office staff or nurses could be intermediaries for medication questions by telephone, participants preferred to speak directly to the doctor. If this was not possible, some participants said that they would schedule an appointment just to ask questions about their medications.

**Figure 1:**
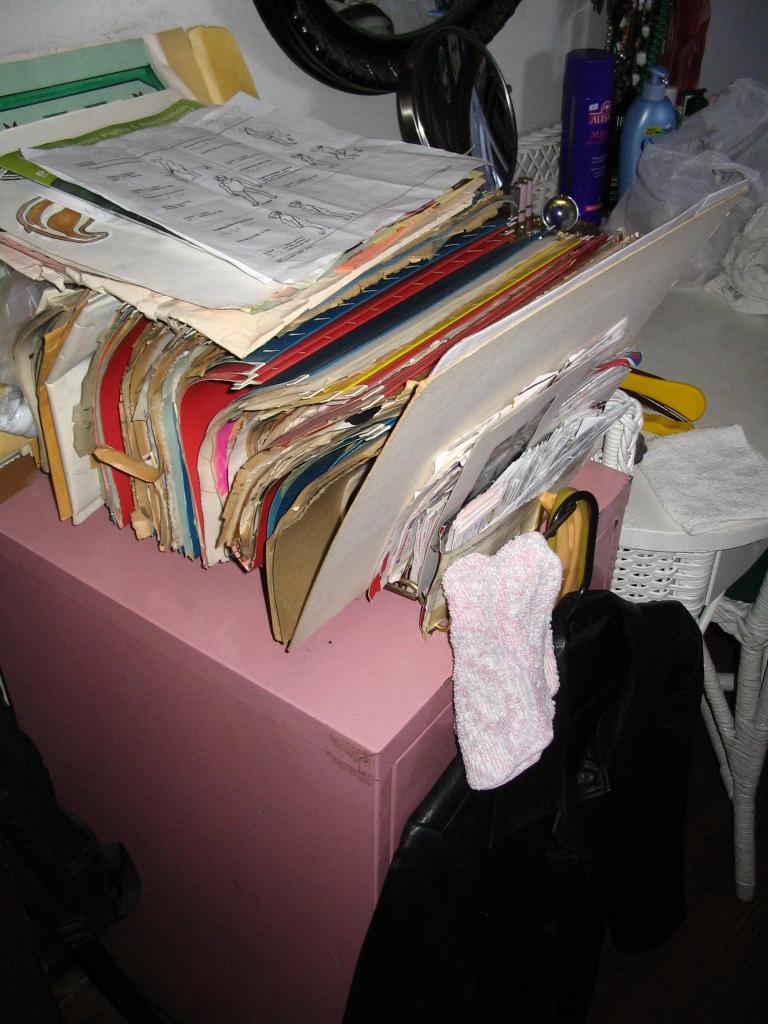
Example of one participant’s personal health information management area

**Figure 2:**
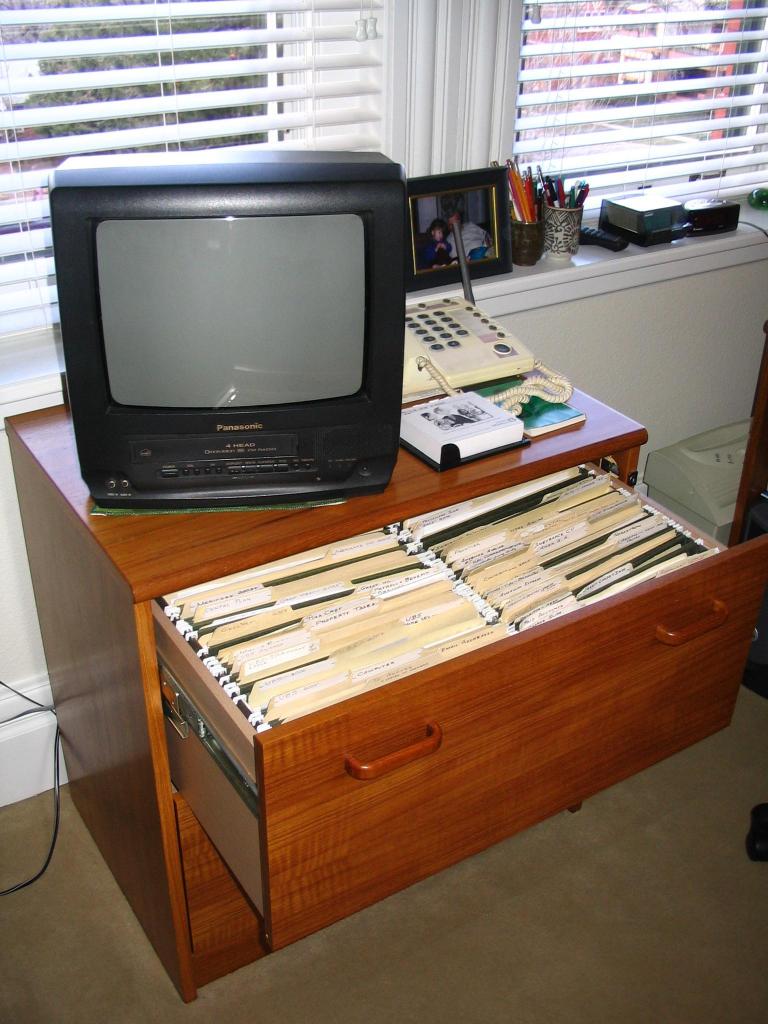
Example of another participant’s personal health information management area

Many participants also used the insert that comes with medications as a resource, but it was most often used when new medications were prescribed, not when questions arose in ongoing medication use. Those who used inserts primarily looked for information on side effects or interactions. Some kept inserts in elaborate files of personal health information (Figure 1, Figure 2), but rarely consulted them after they were filed.

Of the 9 participants who used the Internet, most used it to search for medication information with the Google search engine. The Internet was used for finding quick information, but was usually used in conjunction with other information sources.

Interview participant 1014 A lot of times I ask the pharmacist [questions about my medications] because the pharmacist often times knows some of the connections that maybe the physician is not quite up on ...If I have a real question I would certainly ask my doctor... And as I said, I do go on the Internet if I just want some information. For instance, this doctor had prescribed medication for me and I started taking it and I was disturbed because I woke up in the middle of the night and my mouth was so dry and my throat was so dry and now I was having trouble with urination and I thought you know, that medication is doing this to me. And I’m stopping taking it. So I immediately went on the Internet and even though I had information— well just the brochure that was in it... And I just went on the Internet to find out. And if I ever question something I want to know right away that’s what I do.

Some felt overwhelmed by the number of sources available on the Internet. Others were skeptical about the reliability of websites or did not feel comfortable using the Internet in general.

### Maintaining Autonomy in Medication Treatment Decisions

Participants expressed a desire to remain independent and in control of their medication treatment decisions, despite acknowledging a decline in their mental and physical abilities and sometimes feeling overwhelmed by complicated medication regimens. While some participants followed their doctor’s recommendations without question, others wanted to play a more participatory role in treatment decisions. The latter were more assertive in seeking information, actively monitoring their health, and altering their medication regimens accordingly. For these participants, maintaining autonomy in medication management appeared to be part of a broader desire to maintain autonomy in various aspects of daily life.

Certain medications were considered a threat to autonomy because they were perceived as detrimental to cognition or health. Common reasons participants stopped or altered medication regimens included experiencing negative side effects, feeling they know their own bodily responses to medications (or those of the individual they care for) better than the doctor, or feeling they were on too many medications (a theme that is expanded upon in the next section). When deciding to stop medications, some participants discussed this and negotiated with their doctors while others acted unilaterally. A few made decisions to alter their regimen without consulting with their doctor because they felt their input was not well received in clinic visits.

**Figure 3:**
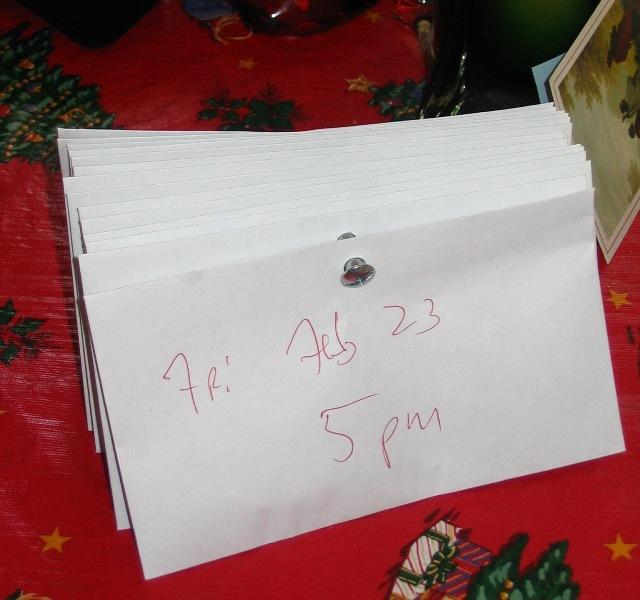
A caregiver created this medication management system to provide some autonomy to her older adult mother. Her mother could access these envelopes fastened together with a pin and pull off the appropriate envelope to receive the correct dosage. This system was also used to carry her mid-day pills with her if she was out of the house

When dosing intervals conflicted with daily activities, the regimen was occasionally perceived as a threat to an individual’s autonomy. Mid-day doses were particularly inconvenient, and several participants mentioned that they frequently forgot them. Others developed elaborate systems with pill boxes, envelopes (Figure 3), or spatial and temporal orderings of medications in multiple locations to integrate medication reminders into everyday life. For instance, participants stored pills in locations where they were likely to come across them at the scheduled dosing time, such as living rooms where they watch television for evening pills, the kitchen for mid-day pills, or the bedroom for nighttime pills.

### Worrying about Taking Too Many Medications

In every interview and focus group we conducted, participants expressed concerns about taking too many medications. They were skeptical of the rationale for and purported benefits of taking multiple medications, particularly for the same condition (eg, hypertension). Most participants desired to reduce the amount of medications they were taking and either approached their doctor, or made unilateral decisions to wean themselves or their loved one from some medications:

Interview participant 1010 I have a lot of hesitancy with respect to medications and I feel like she is getting too much medication and more than likely some of her problems are caused by medication. So I’ve been attempting to modify her medications somewhat. Like weaning her off of prescribed depression medication and using the homeopathic medicine. So in a sense I am being somewhat of a diagnostician and dispenser.

Apprehension about taking too many medications had many dimensions. There was a prevalent view that the potency of medication regimens prescribed to older adults was often inappropriate:

Exploratory focus group 1 participant My biggest frustration with the doctors is overdosing...Here a couple years ago I ended up in the hospital because I was getting bad dizzy spells and I couldn’t breathe and after they checked me out they found out they had overdosed me with medications— the doctor had. So they had to readjust my blood work and medications all over again.

Another common worry was that older adults’ bodies cannot handle multiple medicines:

Confirmatory focus group 2 participant As a caregiver, [I feel that] doctors give too many medications to older people and their bodies— our bodies— can’t process them well and all kinds of disastrous things happen. I’ve seen this many, many times. It’s just too much. The liver doesn’t work like it did when you are younger...It’s just here take this, take this, take this, and then the person suffers.

Participants were concerned about drug interactions and perceived that doctors tended to “layer on” medications, rather than simplifying regimens. Several questioned the concept of taking a medication indefinitely, fearing the medications may overtax vital organs or lead to a dependency. Some participants also voiced concerns about the number of medications they took because, even with medical insurance, it added unnecessary costs to their health care expenses. They felt doctors did not consider cost when prescribing medications.

Although overmedication was the largest area of concern for participants, many did not think their doctors shared their concerns, and as a result the issue was insufficiently addressed. Many shared stories about doctors unwilling to work with them to simplify medication regimens, address their worries, or explain the rationale behind polypharmacy.

### Reconciling Information Discrepancies between Allopathic and Alternative Medical Therapies

Participants consistently distinguished prescription medications from nonprescription medications (eg, vitamins and supplements) as 2 distinct categories. When participants were asked what medications they took, they typically named all prescription medications, and often had to be prompted to list supplements and nonprescription medications. Likewise, nonprescription medications were often separated or omitted from medication lists that they kept on hand and were stored in different locations from prescription medications. Many participants did not feel it necessary to discuss the nonprescription medications they were taking with their doctor. Others had questions about the value of nonprescription medications, vitamins, and supplements in their complex drug regimens, but lacked information or direction because they typically did not think it was appropriate to talk to their doctor about them.

Several participants in the study visited practitioners of alternative forms of health care that included nutritionists, chiropractors, acupuncturists, or homeopaths. Many of these participants were frustrated about conflicting information that they received from their primary care doctor and alternative care providers regarding their health, the etiology of their illnesses, and the safety and effectiveness of their conventional and complementary medication regimens. In general, these participants did not feel that their allopathic doctors supported alternative care and therefore did not always tell them about alternative medications or therapies that they were pursuing. For 2 participants in particular, this was a source of confusion and stress regarding medication management and decision making:

Confirmatory focus group 2 participant The biggest problem in my mind for my personal planning and decision making is the kind of conflict between my primary care doctor, traditional medicine man and this alternative [practitioner] — who happens to be a chiropractor— but he has done a big study of supplements. Consequently I take 20 or 25 pills a day. My primary care physician just doesn’t care a hoot about all those supplements ... Most people do not take all the pills I take, I’ve discovered. They take prescription pills, but I take magnesium and calcium and some brain pills and all kinds of stuff, and those are very important to the alternative medicine person in my life. But they are just uh ... minimized totally by the primary care doctor so I feel like I’m fighting two sides.

Another participant asked:

If you don’t follow the regimen, do you feel any different?

Response:

“Yes, I do...But then I don’t know which one to blame or credit!”

### Tracking and Coordinating Health Information between Multiple Providers

Participants described 2 dimensions of personal responsibility for coordinating health information: first, keeping track of health information through record keeping, creating medication lists, and compiling medication information, and, second, acting as a coordinator through assisting in the transfer of health information between multiple doctors and caregivers. Most kept paper medical files for bills, insurance papers, and medication information inserts. One couple, who split residency between 2 states and had doctors in both, made photocopies of all their medical paperwork and physically moved the papers between states in portable file cabinets. Almost all participants kept medication lists to assist with medical appointment paperwork and in cases of emergency, although some were outdated or illegible.

Although all participants in the study partook in some form of record-keeping behavior, the extent to which participants felt that they needed to review or transfer this information depended largely on whether their multiple doctors were in a closed system. When doctors were outside the umbrella of a single hospital, health maintenance organization, or other network of care, participants did not assume that information would be exchanged automatically or in a timely manner, though they expressed a desire for this to happen. In such cases, participants felt that they needed to take a more proactive role in knowing their health information and acting as a medium between doctors:

Exploratory focus group 2 participant I see two different doctors on a regular schedule and the only thing I really worry about is if one of them changes my medicine I want the other one to know about it because I get medicine from each of them and I don’t want to add something or take away something that is going to cause problems.

Conversely, when doctors did fall within the same network, participants assumed that they were using the same computer system and could view health and medication information from other doctors. In this case, participants tended not to take as prominent a role in managing their health information because they felt confident their records were well maintained, information was being shared, and care was well coordinated.

Personal factors also played a role in the degree to which participants actively coordinated health information. Some participants reported being compulsive about record keeping in general, while others seemed unwilling or unable to actively manage their health information because it was overwhelming or beyond their cognitive abilities.

## Discussion

As part of a user-centered design process for a PHA for older adults, we identified 5 core concerns that older adults have with regard to medication self-management. These findings, many of which corroborate findings from previous studies, suggest functional requirements for PHAs to assist older adults in medication management.

Participants’ desire for reliable medication information is consistent with previous research on consumers’ use of health websites [17], as well as research on keeping certain medication information, such as inserts, in case they are needed [18]. Current PHAs commonly link drug names on a medication list to information about that drug. These links can provide useful information about medication indications and potential side effects, particularly if the information is written for a lay audience (rather than simply replicating the medication insert).

However, more could be done to address older adults’ common latent concerns about overmedication. When polypharmacy is necessary, patients and caregivers want to understand the rationale for it and want to be reassured that long-term use of multiple medications will not damage the body in some way. Current PHAs typically provide information about individual medicines but provide limited information about the medication regimen as a whole. When information on potential drug interactions is provided, the gravity of interactions is unclear. PHAs that allow patients to document troubling symptoms that could indicate side effects and explore whether medications could be the cause may be beneficial in addressing these patient concerns. Ideally, PHAs could help patients assess appropriateness of the drug regimen as a whole through automated analysis or consultative services. While current PHAs focus on electronic messaging with physicians, the ability to share information and receive advice from pharmacists could be even more valuable, since patients find pharmacists to be the most credible resource for medication questions. This is consistent with the vision that PHAs should empower patients by providing new avenues of access to useful and necessary health services [19].

PHAs should also be flexible to accommodate older adults’ desire for control over their regimens. Older adults who self-medicate or experiment with medications to get a desired effect or lessen side effects or lifestyle conflicts are unlikely to be satisfied with a PHA that provides a medication list owned and managed by a physician or practice (as in patient portals, also known as tethered PHRs) [15]. These patients are likely to be more satisfied with a PHA that lets them edit medication lists to reflect changes they have made to their medication regimens. On the other hand, patient portals have the advantage of autopopulating medication information [20]. More flexible interoperable PHAs may need to avoid overtaxing older adults with medication input and reconciliation tasks.

PHAs can also help address the common challenges older adults face in tracking and coordinating care. Previous research has demonstrated the appeal of sharing electronic personal health information among multiple providers to improve care coordination [20,21]. Our findings suggest that PHAs should support care coordination not only among allopathic practitioners and pharmacists, but also with practitioners of complementary medicine. PHAs should allow and encourage nonprescription and alternative medications to be added to medication lists. Authoritative, nonjudgmental information on these medications, and their appropriateness in the medication regimen as a whole, should be addressed. Additionally, because some patients are wary of disclosing their use of alternative medications to allopathic doctors, it may be useful for PHAs to provide patients with the ability to selectively disclose medication information to different practitioners. Given the distinction that older adults and caregivers make between keeping records and coordinating care, PHAs may also need to accommodate multiple views: (1) a more inclusive and comprehensive view for home use, and (2) a more streamlined or compact view appropriate for assessment by practitioners and for mobile use during clinic or emergency visits.

Our findings support and extend previous research demonstrating that medication self-management is a common and challenging issue for older adults and their caregivers [1,3,5]. Strengths of this study include a stepwise analytic approach, with refinement of topic guides based on exploratory focus groups, investigator triangulation in the analysis of transcripts, and the use of focus groups for member checking of findings derived from individual interviews. The research team included expertise in internal medicine, pharmacy, information technology, medical anthropology, and communications. This variety provided the ability to examine data from several perspectives. The qualitative nature of this study also allowed participants to elucidate their primary medication management concerns in an unrestricted manner and gave us more detail about the nature of these concerns and how they could be addressed. The primary limitation of this study is the small sample size, particularly of caregivers. Responses also could have been affected in those instances where caregivers were interviewed with patients as a dyad and when they participated in a focus group with patients [22]. Because of the small sample size and purposive sampling strategy, these findings from participants in the Denver-Boulder metropolitan area may not be fully generalizable to other regions.

This study was undertaken as part of a larger project to develop a prototype interoperable PHA. Our findings have elucidated specific medication management challenges that will inform development of PHAs that are appropriate for the increasingly important but hard to reach older population [20,23]. Since the original qualitative analysis, our team has developed a prototype PHA responsive to these findings [24]. The public release of interoperable platforms to support PHAs that can be tailored for different user populations (eg, Dossia, Google Health, and Microsoft HealthVault) should facilitate development and dissemination of PHAs responsive to the particular needs of older adults.

## Multimedia Appendix 1

Interview guide:

## Multimedia Appendix 2

Participant characteristics survey

## Multimedia Appendix 3

Focus group facilitation guide

## Abbreviations

PHA: personal health application
PHR: personal health record
