# Supplementary material for: Older Adults with Multi-Morbidity: Medication Management Processes and Design Implications for Personal Health Applications
Source: J Med Internet Res. 2011 Jun 29;13(2):e44. doi: 10.2196/jmir.1813 (PMC3221383; doi:10.2196/jmir.1813)
Supplement: Supplementary file 1 [file jmir_v13i2e44_app1.pdf]

Appendix A: Interview Guide
Older Adults with Multi-Morbidity: Medication Management Processes and Design Implications for Personal Health Applications

Thanks for agreeing to talk to us so that we can better understand your health care experiences and how you manage information about your health and health care, including medicines. We're talking to you because you have multiple conditions that you're trying to manage. We're especially interested in learning about your experiences with receiving care from multiple health care providers and your strategies for managing your medications and health care needs.

[If caregiver is present, add] We are interested in the perspectives of both the person who has the multiple medical conditions and the person who has primary responsibility for keeping track of the information needed by health care providers or provided by them. When we ask questions about "managing health or health information" though, we would like to focus on the health and care of the person who has the multiple medical conditions.

To begin with, I'd like to learn a little more about you and become familiar with your health care situation.

1. First, let's talk a little about you. *[Ask questions from participant characteristic's survey. If caregiver is present, get information on them as well.]*

2. Next let's talk about the *current* health conditions you're managing.
    a. What medical problems do you have?
    b. How many different medicines do you take every day?
        i. Do you take others as well that you don't take daily? If so, how many and how often?
        ii. Do you take medicines besides the ones your doctor prescribes for you?
        iii. Do you keep a list of your medications? Do you bring it with you to doctor's visits? Does your doctor find it helpful?
    c. Think of all the health care providers that you have seen in the last year. As I list these different types, can you tell me which of them you have seen?
        - Primary care provider
        - Specialists *[probe for what type of specialists; e.g., eye, skin, cardiologist, etc.]*
        - Surgeons
        - Nurses *[probe for what facet of care they provide; e.g., they may see a diabetes nurse educator on a regular basis]*
        - Dieticians
        - Physical or occupational therapists
        - Other *[acupuncturist, chiropractor or other alternative medicine practitioners]*

Now let's talk about some specific challenges that people with multiple medical conditions often face.

3. I'd like to learn about what it's like living with and trying to manage multiple medical conditions. Tell me about what that's like for you, including what some of the biggest challenges or frustrations are for you. *[Probe for information in the*

following categories as necessary/possible]

a. Coordinating care among doctors
   - *Is it hard to keep track of appointments? How do you do that?*
   - *Do the different doctors you are seeing seem to communicate well?*
   - *Have you run into problems such as one of your doctors not knowing what the other is doing?*

b. Managing multiple medicines
   - *Are your medicines confusing at times? If so, what is confusing about them?*
   - *Have you run into problems with your medicines? If so, what?*

c. Self care and recognizing "red flags"
   - *Do you feel you have a good handle on what you need to do to monitor and take care of your medical problems?*
   - *Are there particular things you look out for – things that mean you should call your doctor or go to the emergency room?*

4. What do you do to coordinate care among all your providers?

5. One challenge we're especially interested in is keeping track of multiple medicines and making sure you're taking the right ones at the right times. I'd be interested to hear how you do that.

   - *If you have questions about your medicines, what do you do to get those questions answered?*
   - *Do you ever visit or call your pharmacist specifically to ask questions or talk about your medications, even when you're not refilling or picking up medications?*
   - *How many pharmacies do you use?*
   - *What has been most helpful to you in keeping track of your medicines? [Probe for people (e.g., doctor, nurse, or pharmacist), written information, other]*
   - *Does your doctor, pharmacist or both explain your new meds if you are on them?*
   - *What information about your medicines is most useful for you?*
   - *Does your doctor give you any written instructions for taking your medicines when he or she prescribes them for you? If not, how do you remember what your doctor tells you after you have left an office visit?*
   - *Do you have to do anything special to get reimbursed for your medication costs? Is it simple or difficult? (If difficult, can you explain?)*
   - *Has there been any trouble or confusion if you have had to go from one brand name medication to another or to a generic, or vice versa?*
   - *Has your doctor given you sample medications? If so, did you keep track of what you took in the same way as you do your regular medications?*
   - *Do you take any medications that require you to have your blood checked or other monitoring on a periodic basis? If so, does the doctor's office remind you to do those checks or do you keep track of that?*

- *What do you do if your doctor cancels a medicine and it is already in your pillbox?*
- *Do you know what each medicine you take looks like?*
- *If you discover you have missed a dose, do you take it later or skip that time usually?*

The next questions are only relevant if you've been hospitalized in the last year. If so, we'll talk more specifically about your hospitalization and your path back home and through recovery. *(If participant has been hospitalized, continue with questions 6 through 17. If not, go to question 18).*

6. What was the reason for your hospitalization?
    a. How long ago was that?
    b. How long did you stay in the hospital?
    c. How long did it take you to fully recover?

7. When you were released from the hospital, did you go directly home or did you stay in other care facilities before returning home?
    [If intermediate facilities]
    a. What were those other care facilities, and how long did you stay in those?
    b. What kind of care were you receiving in those other facilities?
    c. *[Probe for sequence and timing]*

8. Did any friends, family members, or health care workers help you while you were recovering?
    a. If so, who helped and in what ways?
    b. Do you still receive any health care help from these people? If so, what kind?

9. Before you learned that you needed to go into the hospital, which health care provider(s) were you seeing on a regular basis?

10. What new or additional providers became involved in your care as the result of the condition that caused the need for you to go into the hospital?

11. *[Posed to caregiver]* Did your caregiving role or responsibilities for managing health information change as a result of *[index person's]* hospitalization?

We're interested in *new* health management challenges that you encountered during the transition in care that you experienced.

12. What were the biggest challenges for you during the transition?

13. Did your health care providers ask for information from you that you had not anticipated?

14. Were you able to provide all the information you were asked to provide?
    a. If not, what information did you find hard to provide?

15. Were you able to keep track of all the information the health care providers gave you?
    a. If not, what made it difficult for you to keep track of everything?

16. What might have made the transition experience easier for you?

17. Based on your transition experience, did you make any changes to the way you had been managing your health information before your hospitalization?
    a. If so, what changes did you make?

We're also interested in how you manage information about your health and health care. It would be most helpful, as we talk about this, if you can show us where you keep your health information, how you organize and store it, and ways you have of knowing how to find information you need when you need it.

HEALTH INFORMATION MANAGEMENT

18. Do you have your medical information written down anywhere?
    a. What information do you have written down?
    b. Has it been useful for you to keep that information?
    c. In what ways is it useful to you?

19. Do you keep track of your appointments?
    a. If so, how do you do that?
    b. If not, why not?

20. Is there any of that information that you take with you to appointments with your doctor or other health care professionals?
    a. Under what circumstances do you take it with you?
    b. Which information do you take with you?

21. Where do you keep your health information?
    a. How do you make sure you have what you would need in an emergency?

22. Do you keep books or other reference materials, such as pamphlets, that you use to help you make decisions about your health care?

MEDICATIONS

23. Do you keep track of your medicines?
    a. If so, what information about your medicines do you keep?
    b. How do you do that?
    c. If not, why not?

24. Where do you keep your medicines?

25. Do you keep any old medicines on hand – medicines that you are not currently taking?

Appendix A: Interview Guide
Older Adults with Multi-Morbidity: Medication Management Processes and Design Implications for Personal Health Applications

26. How do you keep track of what to take and when?

FOR COMPUTER USERS

Computer uses for managing health information

27. Do you use a computer in any way to help manage health information *[your] or* [*index person's*] health care?

IF YES

a. If so, how did you use it?
b. Can you tell me what worked for you and what didn't?

IF NO

c. What has prevented you from using the computer for this purpose?

28. Do you have any suggestions or ideas on how a computer might have helped you manage health information or otherwise helped you during the transition experience?

FOR SELF-ONLY CARE INDEX INDIVIDUALS

29. When you hit snags with regard to managing your health needs (e.g. can't get answers, can't get what they need from health care providers), do you seek help from others?
    a. If so, who do you go to for help?

IN-HOME CAREGIVER

30. Do you do anything special to manage the health information for other household members (especially the index person) that is different from what you do with your own health information?
    a. If so, what and why?

31. Can you tell me about challenges specific to managing health information for a person other than yourself?

LOCAL CAREGIVER

32. Can you tell me about any challenges that you feel are particular to managing health information for a person who doesn't live with you?
