# Supplementary material for: Older Adults with Multi-Morbidity: Medication Management Processes and Design Implications for Personal Health Applications
Source: J Med Internet Res. 2011 Jun 29;13(2):e44. doi: 10.2196/jmir.1813 (PMC3221383; doi:10.2196/jmir.1813)
Supplement: Supplementary file 2 [file jmir_v13i2e44_app2.pdf]

APPENDIX B: PARTICIPANT CHARACTERISTICS SURVEY

Participant ID# ____________________
Date: __________________________
Start time: __________
End time: ___________
Recorder: __________________________

DEMOGRAPHICS

1. Age: __________

2. Sex: Male ☐ Female ☐

3. Race/Ethnicity: *(as identified by participant)*
   ____

4. Marital status: Married ☐ Single (unmarried, widowed or divorced) ☐

5. Ages of other members of the same household:

   Spouse _________ Children ________________ Others ______________

   [If possible, find out relationships of "others" to index individual]

PERCEIVED HEALTH STATUS

6. In general, would you say that your health is:

   ☐ Excellent
   ☐ Very good
   ☐ Good
   ☐ Fair
   ☐ Poor

COMPUTER USE/SKILLS *(pose to person who manages <u>all or some</u> of their own health information)*

7. Have you ever used a computer? Yes ☐ No ☐ *If NO, go to #8; If YES, go to #10*

8. Are you curious about using computers or interested in trying? Yes ☐ No ☐

   If NO, go to #9;
   If YES, go to #10

9. Do you mind sharing why you're not? *(Don't press if participant wishes not to respond.)*

   After response, END

10. Do you have access to a computer? Yes ☐ No ☐ *If NO, go to #18; If YES, go to #11*

11. Where is that computer located? (e.g., in the home, at the library, a friend or family member's home, etc.)

12. Is that computer connected to the Internet? Yes ☐ No ☐ Don't know ☐

13. Do you ever use that computer? Yes ☐ No ☐ *If NO, go to #14; If YES, go to #15*

14. Do you mind sharing why you don't? (*Don't press if participant wishes not to respond.)*

    After response, go to #18

15. How often do you use that computer? *(Probe to find out hour many minutes/ hours per time they use it)*

16. What applications or programs do you use? *(e.g., games, financial, word processing, E-mail, etc.)*

17. How often do you use ____________________? *(Ask about each application they list)*

18. Have you ever used the Internet? Yes ☐ No ☐ *If NO, END; If YES, go to #19*

19. Do you currently use the Internet? Yes ☐ No ☐ *If NO, go to #21; If YES, go to #20*

20. How often do you use the Internet?

21. What do you do (or have you done) on the Internet?
