# Supplementary material for: Older Adults with Multi-Morbidity: Medication Management Processes and Design Implications for Personal Health Applications
Source: J Med Internet Res. 2011 Jun 29;13(2):e44. doi: 10.2196/jmir.1813 (PMC3221383; doi:10.2196/jmir.1813)
Supplement: Supplementary file 3 [file jmir_v13i2e44_app3.pdf]

APPENDIX C: FOCUS GROUP FACILITATION GUIDE

Welcome

Hello to everyone, welcome and thank you for agreeing to be part of our focus group today.

Introductions

First of all, let me introduce our team: I'm (name of facilitator) and this is (name of co-facilitator). We're working on a project with the University of Colorado in collaboration with University of Colorado Hospital.

Exploratory Focus Groups: We are interested in hearing about how you manage your health information or help someone else manage their health information, such as medications, appointments and medical problems. We will be asking questions about your experiences in the health care system in general, about how you manage or help someone else manage their health information, and about how you manage medications.

Verification Focus Groups: We have been talking to people about how they manage their health information or help someone else manage their health information, such as medications, appointments and medical problems. Some of you have participated in the individual or group interviews that are the basis for what we are about to do. Today we would like to review findings from those interviews with you to verify our understanding of what was heard, and make sure we also know what issues are most important.

First we will be asking you to comment on how well these findings represent your reality – what you know and experience every day.   You may tell us that some things are missing, or that some are not really an issue. Following that, we will do an exercise to help us prioritize the issues and needs that you verified to be accurate.

Both Focus Groups: You've been invited here today to give your opinion as a person who may have multiple medical conditions and manages your own health information, or because you help someone else manage their health information. You've also been invited to participate if you've been hospitalized recently or have helped someone else who was hospitalized and came home to recover. Today we'd like you to join our discussion as a person who has experienced such things. We know you may have other roles in the community and in your daily life, but today we would like your views as a person who has managed health information or medications, rather than from the perspective of your other roles. We value your opinions and want you to know that we hope to use the information in to learn more about important health issues and needs in regards to managing multiple medications and in managing health information.

Ground rules

Before we begin, let me mention a few things about how we usually conduct these groups:

1) I will be the facilitator for the group. My role is to ask the questions we have for the group, and to encourage everyone to participate. I won't be doing much talking, but may ask you to explain more or to give an example. Also, it's my job to see that everyone has a chance to voice their opinions, as well as to keep us moving along so that we have time to discuss all of the questions. So, at times, it might seem as though I am cutting you off, and this is not meant to be rude but rather to make sure that we have time to hear from everyone on each question. Since we only have until (STATE TIME HERE), we won't have time to hear many details of each person's situation. We know that you have each been through your own experience and that sharing your experience with others can be helpful. We hope you'll understand that for these next 1½ hours we will ask you to focus on the questions asked. You can take extra time after the group is finished to talk more with each other if you wish. We want to thank each of you for being here, so please know that we value your ideas and comments.

2) It's really important that everyone hear this: THERE ARE NO RIGHT OR WRONG ANSWERS! Each person's experiences and opinions are valid, and we want to hear a wide range of opinions on the questions we'll be asking. So, please speak up, whether you agree or disagree with what's being said, and let us know what you think.

3) Sometimes participants bring up sensitive issues during these discussions, and we want to be sure that everyone agrees before we begin the group that anything of a personal nature that is mentioned in this room will NOT be repeated to others outside of this discussion group. Can I see a nod from everyone showing me that you agree with this confidentiality ground rule? (If anyone is not willing to give their consent to confidentiality, they may be excused from the group.)

4) Let me tell you about our recording process. As you can see, we have a tape recorder today. We usually record these focus groups because we want to get everything that all of you say, and we simply can't write fast enough to get it all down. We use first names only in the transcript, and when we put together the results from all the groups, we don't include any names.

   It is VERY IMPORTANT that we speak ONE AT A TIME, so that we have a good quality recording. So, now that you know what our process is, is everyone OK with being recorded?

5) Let me mention before we start, that we plan to be finished with our discussion by STATE END TIME. After our discussion, you will receive the gift cards for King Soopers as our thank-you to you for participating in our

group tonight.

6) In case anyone needs to use the restroom, they are located ___________. One last thing, we ask that everyone turn their cell phones off or to silent mode so that we can begin our discussion. Thanks.

Exploratory Focus Group Questions

1. Let's start by going around the room to introduce ourselves. Will each person please tell us:
    - Your first name
    - How long you've lived [in your neighborhood]
    - [*Ice-breaker question*] What your favorite activity is at [location where group is conducted].

2. Now we're going to talk about your experiences with managing multiple medical conditions – or maybe for some of you, about your experience with returning back home and recovering after being hospitalized. Has anyone been hospitalized or helped take care of someone who has been hospitalized within the last year?
    - *If anyone indicates so, acknowledge it and encourage them to share information about that experience as we proceed.*

    a. What are some of your *biggest frustrations* with trying to manage multiple medical conditions, which may require you to see different doctors, take a lot of different medicines, have different tests done periodically, and so forth?
    Probes:

    i. Is it hard to keep track of appointments, to know when to have tests done, or to know when to follow up with your doctors? How do you do that?
    ii. Do you ever have trouble getting information that you want from your doctor's office when you need it? What do you do when you can't?
    iii. Do you ever have the impression that one of your doctors doesn't know what another one is doing? What do you do when that happens?
    iv. Do you ever wonder where to find good information that you can trust about your health condition or medicines? How do you find that information when you need it?

    b. [*Pose to people who indicated they have experienced a major transition in the last year*] What were some of your *biggest frustrations* with trying to manage the transition from the hospital back home, and to recover from the illness that put you [or your friend/family member] in the hospital?
    Probes:

i. Did you ever find yourself confused or uncertain about what you should be doing during your recovery? What did you do to reconcile that?
ii. What might have made the transition experience easier for you?
iii. Do you do anything differently now in terms of managing your health and health information than you did before you had this experience?

3. One challenge we're especially interested in is keeping track of multiple medicines and taking the right ones at the right times. We'd like to hear how you do that.
   a. Tell me how you get the information you need to take your medications safely.
      Probes:

      i. What information source(s) do you trust the most?
      ii. What do you do when you can't get the information you need?

   b. How do you keep track of your medicines?
      Probe:

      i. What or who has been most helpful to you in keeping track of your medicines?
      ii. What tools or techniques do you use to remind yourself when to take your medicines, how much to take, when to refill them, which ones are current and not, and so forth?

   c. Can you tell us about any experiences you've had when your medicines were confusing?
      Probes:

      i. What caused the confusion?
      ii. What did you do about that?

   d. Are there any other thoughts about managing medicines that we haven't already covered that you you'd like us to know about?

4. Your doctor's office, your insurance company, and your pharmacy already have a lot of information about you stored electronically. We'd like to hear your thoughts about how you might like to see your personal health information handled.

   a. Would you like to have access to that information?

   b. What information would you be interested in having access to?

   c. If you could carry that information electronically and securely from one doctor to another on something as small as a credit card, for

example, how would you feel about using that?

d. What concerns would you have about that?

5. Is there anything we've missed? Anything else we should know?
   Thanks so much for being here today and for sharing your ideas with us!

Verification Focus Group Questions

1. Let's start by going around the room to introduce ourselves. Will each person please tell us:
   - Your first name
   - How long you've lived [in your neighborhood]
   - [*Ice-breaker question*] What your favorite activity is at [the location the group is conducted].

2. First we're going to review the **goals** that people told us about – the things about managing their health information and especially their medications that are most important to them. As I read through these goals, I will ask for your comment:
   - Does this ring true for you? Does it seem to reflect what is important to you or to people you know?
   - If not, what would be a more accurate way to describe it?
   - Is there something missing or something you can add that will help us understand this better?

   Some types of goals include, but are not limited to:

   - *Maintain my independence*
   - *(For caregivers) Ensure that my loved one can remain home rather than be placed in a care facility*
   - *Ensure safe, effective use of my medications*
   - *Be able to work more effectively with my doctor to manage my health*
   - *Maintain my active lifestyle while managing my medical conditions responsibly*

3. Now we're going to talk about the **tasks** that people said they needed to perform to manage multiple medical conditions, which may require them to see different doctors, take a lot of different medicines, have different tests done periodically, and so forth. As I read through this list of tasks, I will ask for your comment:

   - Does this sound familiar? Does it seem to reflect your experience or that of people you know?
   - If not, what would be a more accurate way to describe it?
   - Is there something missing or something you can add that will help us understand this better?

   Some tasks include, but are not limited to:

- *Keeping/updating medication list*
- *Pill taking (e.g., remembering when to take, when last dose taken)*
- *Managing pill scheduling/organization (reviewing reported strategies, such as pillboxes, and underlying logic)*
- *Reflection of medication regimen (e.g., assessing potential side effects, weighing cost alternatives)*
- *Monitoring medication-related parameters (e.g., tracking self-monitoring, such as blood sugar levels related to insulin doses, or lab monitoring, such as INR related to Coumadin dose)*
- *Purchasing and refilling medications*

4. Now we're going to ask you to help us understand how important the tasks are relative to one another. You'll do this by putting a sticker next to your "top 5" tasks. You can put more than one sticker next to a task if you think that one deserves more of your 5 votes than others. You can even give all 5 votes to one if you want! After everyone is finished, we'll count these up and see what the group came up with and see if you have any further thoughts to share about those.

5. Your doctor's office, your insurance company, and your pharmacy already have a lot of information about you stored electronically. We'd like to hear your thoughts about how you might like to see your personal health information handled.

    a. Would you like to have access to that information?

    b. What information would you be interested in having access to?

    c. If you could carry that information electronically and securely from one doctor to another on something as small as a credit card, for example, how would you feel about using that?

    d. What concerns would you have about that?

6. Is there anything we've missed? Anything else we should know?

Thanks so much for being here today and for sharing your ideas with us!
